# Supplementary material for: Depression and Anxiety in Association with Polypharmacy in Patients with Multiple Sclerosis
Source: J Clin Med. 2023 Aug 18;12(16):5379. doi: 10.3390/jcm12165379 (PMC10456074; doi:10.3390/jcm12165379)
Supplement: Supplementary file 1 [file jcm-12-05379-s001.zip › Supplementary Document S1.pdf]

Supplementary Document S1: Structured interview

Baseline questionnaire – 2019

Sociodemographic

Birth date: _____years

Sex: ☐ female
☐ male

Partnership status: ☐ single living
☐ partnership

Current job situation:
☐ in training
☐ studies
☐ employment: ☐ full-time ☐ part-time
☐ unemployment
☐ pension: ☐ due to old age ☐ due to disability
☐ other

School years (at most until A-levels): _____years

Educational level: ☐ no training
☐ skilled worker
☐ technical college
☐ university

Place of residence:

____
☐ rural community (until 5000 inhabitants)
☐ provincial town (>5000-20000 inhabitants)
☐ medium-sized town (>20000-100000 inhabitants)
☐ city (>100000 inhabitants)

Number of children: ____

Number of siblings: ____

Clinical-neurological

Current pregnancy: ☐ no
☐ yes

Pregnancy planned in the near future: ☐ no
☐ yes

Number and kind of comorbidities:

Disease course: ☐ clinically isolated syndrome (CIS)
☐ relapsing-remitting multiple sclerosis (RRMS)
☐ secondary progressive multiple sclerosis (SPMS)
☐ primary progressive multiple sclerosis (PPMS)
☐ undefined

Clinical care: ☐ outpatient
☐ inpatient

Date of initial CIS/MS diagnosis (month/year): ____

Current Expanded Disability Status Scale (EDSS) Score: ____

Medication

Use of disease-modifying drugs (DMD)

	Currently	Previously	Never	Unknown
Interferon beta (Avonex, Rebif, Plegridy, Betaferon, Extavia)	☐	☐	☐	☐
Glatiramer acetate (Copaxone)	☐	☐	☐	☐
Teriflunomide (Aubagio)	☐	☐	☐	☐
Fingolimod (Gilenya)	☐	☐	☐	☐
Dimethyl fumarate (Tecfidera)	☐	☐	☐	☐
Mitoxantrone (Novantron, Ralenova)	☐	☐	☐	☐
Natalizumab (Tysabri)	☐	☐	☐	☐
Alemtuzumab (Lemtrada)	☐	☐	☐	☐
Daclizumab	☐	☐	☐	☐
Ocrelizumab (Ocrevus)	☐	☐	☐	☐
Cladribine (Mavenclad)	☐	☐	☐	☐
Intravenous immunoglobulin G (Octagam)	☐	☐	☐	☐
Azathioprine (AZA-effect, Aza-Q, Azafalk, Azaimun, Azamedac, Imurek, Imurel)	☐	☐	☐	☐
Cyclophosphamide	☐	☐	☐	☐
Cyclic glucocorticosteroids (Urbason)	☐	☐	☐	☐

Reason for DMD switch:

☐ Side effects
☐ Disease activity despite DMD treatment
☐ Pregnancy
☐ Personal
☐ Other

Medication schedule

	Preparation	Drug	Indication	Dosage	Intake interval	Route of administration	Prescription status	Therapy goal (DMD, symptom treatment, treatment of comorbidities or other conditions)
1								
2								
3								
4								
5								
6								
7								
8								
9								
10								
11								
12								
13								
14								
15								
16								
17								
18								
19								
20								
