# Supplementary material for: Depression and Anxiety in Association with Polypharmacy in Patients with Multiple Sclerosis
Source: J Clin Med. 2023 Aug 18;12(16):5379. doi: 10.3390/jcm12165379 (PMC10456074; doi:10.3390/jcm12165379)
Supplement: Supplementary file 1 [file jcm-12-05379-s001.zip › Supplementary Table S1.pdf]

Supplementary Table S1: Frequency of use of drug classes according to ATC classification system in relation to anxiety (*n* (%))

	HADS-A 0-7	HADS-A 8-10	HADS-A 11-21	Total sample	*p*
A02 - Drugs for acid related disorders	49 (23.7)	25 (24.3)	15 (23.4)	89 (23.8)	0.990
A03 - Drugs for functional gastrointestinal disorders	0 (0.0)	1 (1.0)	1 (1.6)	2 (0.5)	0.253
A04 - Antiemetics and antinauseants	0 (0.0)	1 (1.0)	0 (0.0)	1 (0.3)	0.267
A06 - Drugs for constipation	5 (2.4)	3 (2.9)	0 (0.0)	8 (2.1)	0.413
A07- Antidiarrheals, intestinal anti-inflammatory/anti-infective agents	2 (1.0)	3 (2.9)	0 (0.0)	5 (1.3)	0.221
A10 - Drugs used in diabetes	7 (3.4)	3 (2.9)	4 (6.3)	14 (3.7)	0.499
A11 – Vitamins	100 (48.3)	52 (50.5)	35 (54.7)	187 (50.0)	0.667
A12 - Mineral supplements	23 (11.1)	18 (17.5)	9 (14.1)	50 (13.4)	0.296
A16 - Other alimentary tract and metabolism products	0 (0.0)	1 (1.0)	0 (0.0)	1 (0.3)	0.267
B01 - Antithrombotic agents	33 (15.9)	15 (14.6)	5 (7.8)	53 (14.2)	0.263
C01- Cardiac therapy	3 (1.4)	2 (1.9)	1 (1.6)	6 (1.6)	0.948
C02 - Antihypertensive drugs	1 (0.5)	2 (1.9)	0 (0.0)	3 (0.8)	0.292
C03 - Diuretic drugs	10 (4.8)	4 (3.9)	3 (4.7)	17 (4.5)	0.930
C07 - Beta blocking agents	23 (11.1)	14 (13.6)	11 (17.2)	48 (12.8)	0.430
C08 - Calcium channel blockers	10 (4.8)	4 (3.9)	2 (3.1)	16 (4.3)	0.818
C09 - Agents acting on the renin-angiotensin system	43 (20.8)	15 (14.6)	8 (12.5)	66 (17.6)	0.199
C10 - Lipid modifying agents	22 (10.6)	12 (11.7)	5 (7.8)	39 (10.4)	0.725
G04 - Urological drugs	40 (19.3)	13 (12.6)	7 (10.9)	60 (16.0)	0.150
H02 - Corticosteroids systemic	43 (20.8)	19 (18.4)	9 (14.1)	71 (19.0)	0.482
H03 - Thyroid therapy	30 (14.5)	18 (17.5)	5 (7.8)	53 (14.2)	0.215
L02 - Endocrine therapy	1 (0.5)	0 (0.0)	2 (3.1)	3 (0.8)	0.066
L03 - Immunostimulants drugs	47 (22.7)	28 (27.2)	12 (18.8)	87 (23.3)	0.437
L04 - Immunosuppressants drugs	76 (36.7)	38 (36.9)	26 (40.6)	140 (37.4)	0.845
M01 - Anti-inflammatory and antirheumatic drugs	35 (16.9)	21 (20.4)	11 (17.2)	67 (17.9)	0.743
M02 - Topical products for joint and muscular pain	0 (0.0)	1 (1.0)	0 (0.0)	1 (0.3)	0.267
M04 - Antigout preparations	1 (0.5)	1 (1.0)	1 (1.6)	3 (0.8)	0.682
M05 - Drugs effecting bone structure and mineralization	5 (2.4)	2 (1.9)	0 (0.0)	7 (1.9)	0.459
M09 - Other drugs for disorders of the musculo-skeletal system	2 (1.0)	0 (0.0)	0 (0.0)	2 (0.5)	0.444
N02 - Analgesic drugs	52 (25.1)	27 (26.2)	23 (35.9)	102 (27.3)	0.227
N03 - Antiepileptic drugs	28 (13.5)	14 (13.6)	8 (12.5)	50 (13.4)	0.975
N04 - Antiparkinson drugs	2 (1.0)	4 (3.9)	5 (7.8)	11 (2.9)	**0.014**
N05 - Psycholeptic drugs	3 (1.4)	3 (2.9)	7 (10.9)	13 (3.5)	**0.001***
N06 – Psychoanaleptics	28 (13.5)	25 (24.3)	19 (29.7)	72 (19.3)	**0.005**
N07 - Other nervous system drugs	20 (9.7)	9 (8.7)	3 (4.7)	32 (8.6)	0.460
R03 - Drugs for obstructive airway diseases	6 (2.9)	6 (5.8)	2 (3.1)	14 (3.7)	0.424
R05 - Cough and cold drugs	1 (0.5)	0 (0.0)	0 (0.0)	1 (0.3)	0.667
R06 - Antihistamines for systemic use	15 (7.2)	7 (6.8)	6 (9.4)	28 (7.5)	0.812
V03 - All other therapeutic products	0 (0.0)	0 (0.0)	2 (3.1)	2 (0.5)	**0.008**
V06 - General nutrients	10 (4.8)	7 (6.8)	8 (12.5)	25 (6.7)	0.100
V60A – Homeopathics	3 (1.4)	1 (1.0)	3 (4.7)	7 (1.9)	0.181

Chi-square test was used to calculate p-values, *– *p* after FDR correction <0.05, df – degree of freedom, FDR – False Discovery Rate, HADS-A – subscale of anxiety of the Hospital Anxiety and Depression Scale, *n* – number of patients, *p* – p-value for comparing patients with different HADS-A score
