# Supplementary material for: Depression and Anxiety in Association with Polypharmacy in Patients with Multiple Sclerosis
Source: J Clin Med. 2023 Aug 18;12(16):5379. doi: 10.3390/jcm12165379 (PMC10456074; doi:10.3390/jcm12165379)
Supplement: Supplementary file 1 [file jcm-12-05379-s001.zip › Supplementary Table S2.pdf]

Supplementary Table S2. Frequency of use of drug classes according to ATC classification system in relation to depression (*n* (%))

	HADS-D 0-7	HADS-D 8-10	HADS-D 11-21	Total sample	*p*
A02 - Drugs for acid related disorders	54 (20.5)	23 (32.4)	12 (31.6)	89 (23.9)	0.056
A03 - Drugs for functional gastrointestinal disorders	0 (0.0)	2 (2.8)	0 (0.0)	2 (0.5)	**0.014**
A04 - Antiemetics and antinauseants	1 (0.4)	0 (0.0)	0 (0.0)	1 (0.3)	0.813
A06 - Drugs for constipation	6 (2.3)	2 (2.8)	0 (0.0)	8 (2.1)	0.605
A07 - Antidiarrheals, intestinal anti-inflammatory/anti-infective agents	4 (1.5)	0 (0.0)	1 (2.6)	5 (1.3)	0.471
A10 - Drugs used in diabetes	5 (1.9)	8 (11.3)	1 (2.6)	14 (3.8)	**0.001***
A11 – Vitamins	129 (48.9)	37 (52.1)	20 (52.6)	186 (49.9)	0.833
A12 - Mineral supplements	34 (12.9)	13 (18.3)	3 (7.9)	50 (13.4)	0.282
A16 - Other alimentary tract and metabolism products	0 (0.0)	1 (1.4)	0 (0.0)	1 (0.3)	0.119
B01 - Antithrombotic agents	37 (14.0)	14 (19.7)	2 (5.3)	53 (14.2)	0.118
C01- Cardiac therapy	2 (0.8)	3 (4.2)	1 (2.6)	6 (1.6)	0.104
C02 - Antihypertensive drugs	2 (0.8)	1 (1.4)	0 (0.0)	3 (0.8)	0.726
C03 - Diuretic drugs	8 (3.0)	8 (11.3)	1 (2.6)	17 (4.6)	**0.011**
C07 - Beta blocking agents	32 (12.1)	12 (16.9)	4 (10.5)	48 (12.9)	0.510
C08 - Calcium channel blockers	12 (4.5)	2 (2.8)	2 (5.3)	16 (4.3)	0.777
C09 - Agents acting on the renin-angiotensin system	47 (17.8)	14 (19.7)	5 (13.2)	66 (17.7)	0.691
C10 - Lipid modifying agents	24 (9.1)	14 (19.7)	1 (2.6)	39 (10.5)	**0.009**
G04 - Urological drugs	40 (15.2)	13 (18.3)	7 (18.4)	60 (16.1)	0.747
H02 - Corticosteroids systemic	45 (17.0)	19 (26.8)	7 (18.4)	71 (19.0)	0.179
H03 - Thyroid therapy	37 (14.0)	13 (18.3)	3 (7.9)	53 (14.2)	0.328
L02 - Endocrine therapy	1 (0.4)	1 (1.4)	1 (2.6)	3 (0.8)	0.284
L03 - Immunostimulants drugs	67 (25.4)	10 (14.1)	10 (26.3)	87 (23.3)	0.122
L04 - Immunosuppressants drugs	101 (38.3)	23 (32.4)	15 (39.5)	139 (37.3)	0.634
M01 - Anti-inflammatory and antirheumatic drugs	44 (16.7)	12 (16.9)	11 (28.9)	67 (18.0)	0.177
M02 - Topical products for joint and muscular pain	1 (0.4)	0 (0.0)	0 (0.0)	1 (0.3)	0.813
M04 - Antigout preparations	1 (0.4)	2 (2.8)	0 (0.0)	3 (0.8)	0.105
M05 - Drugs effecting bone structure and mineralization	5 (1.9)	2 (2.8)	0 (0.0)	7 (1.9)	0.586
M09 - Other drugs for disorders of the musculo-skeletal system	2 (0.8)	0 (0.0)	0 (0.0)	2 (0.5)	0.660
N02 - Analgesic drugs	65 (24.6)	26 (36.6)	11 (28.9)	102 (27.3)	0.128
N03 - Antiepileptic drugs	28 (10.6)	12 (16.9)	10 (26.3)	50 (13.4)	**0.018**
N04 - Antiparkinson drugs	4 (1.5)	4 (5.6)	3 (7.9)	11 (2.9)	**0.031**
N05 - Psycholeptic drugs	5 (1.9)	3 (4.2)	5 (13.2)	13 (3.5)	**0.002**
N06 – Psychoanaleptics	40 (15.2)	17 (23.9)	15 (39.5)	72 (19.3)	**<0.001***
N07 - Other nervous system drugs	17 (6.4)	9 (12.7)	6 (15.8)	32 (8.6)	0.061
R03 - Drugs for obstructive airway diseases	11 (4.2)	2 (2.8)	1 (2.6)	14 (3.8)	0.807
R05 - Cough and cold drugs	1 (0.4)	0 (0.0)	0 (0.0)	1 (0.3)	0.813
R06 - Antihistamines for systemic use	17 (6.4)	6 (8.5)	5 (13.2)	28 (7.5)	0.321
V03 - All other therapeutic products	0 (0.0)	2 (2.8)	0 (0.0)	2 (0.5)	**0.014**
V06 - General nutrients	14 (5.3)	7 (9.9)	4 (10.5)	25 (6.7)	0.241
V60A – Homeopathics	4 (1.5)	2 (2.8)	0 (0.0)	6 (1.6)	0.524

Chi-square test was used to calculate p-values, *– *p* after FDR correction <0.05, df – degree of freedom, FDR – False Discovery Rate, HADS-D – subscale of depression of the Hospital Anxiety and Depression Scale, *n* – number of patients, *p* – p-value for comparing patients with different HADS-D score
