# Supplementary material for: Depression and Anxiety in Association with Polypharmacy in Patients with Multiple Sclerosis
Source: J Clin Med. 2023 Aug 18;12(16):5379. doi: 10.3390/jcm12165379 (PMC10456074; doi:10.3390/jcm12165379)
Supplement: Supplementary file 1 [file jcm-12-05379-s001.zip › Supplementary Table S3.pdf]

Supplementary Table S3. The most common comorbidities occurring besides MS stratified by anxiety and depression severity level (*n* (%))

	HADS-A				HADS-D			
	0-7	8-10	11-21	*p*	0-7	8-10	11-21	*p*
Hypertension	55 (26.6)	24 (23.3)	14 (21.9)	0.683	64 (24.2)	21 (29.6)	8 (21.1)	0.551
Depression	18 (8.7)	23 (22.3)	19 (29.7)	**<0.001*****	30 (11.4)	14 (19.7)	16 (42.1)	**<0.001*****
Thyroid disease	34 (16.4)	17 (16.5)	6 (9.4)	0.358	39 (14.8)	16 (22.5)	2 (5.3)	0.053
Symptoms of nutrient deficiency	28 (13.5)	12 (11.7)	4 (6.3)	0.287	32 (12.1)	11 (15.5)	1 (2.6)	0.134
Gastrointestinal symptoms	17 (8.2)	11 (10.7)	8 (12.5)	0.545	23 (8.7)	6 (8.5)	7 (18.4)	0.154
Dyslipidemia	17 (8.2)	7 (6.8)	3 (4.7)	0.623	17 (6.4)	9 (12.7)	1 (2.6)	0.101
Bladder symptoms	12 (5.8)	9 (8.7)	3 (4.7)	0.503	15 (5.7)	5 (7.0)	4 (10.5)	0.509
Osteoporosis	12 (5.8)	8 (7.8)	1 (1.6)	0.235	16 (6.1)	4 (5.6)	1 (2.6)	0.692
Migraine	3 (1.4)	6 (5.8)	8 (12.5)	**0.001****	11 (4.2)	5 (7.0)	1 (2.6)	0.491
Allergy	12 (5.8)	4 (3.9)	1 (1.6)	0.339	17 (6.4)	0 (0.0)	0 (0.0)	**0.025**
Diabetes mellitus	8 (3.9)	4 (3.9)	4 (6.3)	0.693	7 (2.7)	8 (11.3)	1 (2.6)	**0.006***
Asthma bronchiale	9 (4.3)	5 (4.9)	3 (4.7)	0.978	13 (4.9)	2 (2.8)	2 (5.3)	0.734

Chi-square test was used to calculate p-values, *- *p* after FDR correction <0.05, ***p* after FDR correction <0.01, *** *p* after FDR correction <0.001, df – degree of freedom, FDR – False Discovery Rate, HADS-A – subscale of anxiety of the Hospital Anxiety and Depression Scale, HADS-D – subscale of depression of the Hospital Anxiety and Depression Scale, *n* – number of patients, *p* – p-value for comparing patients with different HADS-A or HADS-D score
